# Arsenic Contamination of Ground Water and its Health Impact on Population of District of Nadia, West Bengal, India

**DOI:** 10.4103/0970-0218.66897

**Published:** 2010-04

**Authors:** Debendra Nath Guha Mazumder, Aloke Ghosh, Kunal Kanti Majumdar, Nilima Ghosh, Chandan Saha, Rathindra Nath Guha Mazumder

**Affiliations:** DNGM Research Foundation, 37/C Block B New Alipore, Kolkata - 700 053, India; 1 Department of Community Medicine, KPC Medical College, Kolkata, India; 2 Department of Chemistry, School of Tropical Medicine, C. R. Avenue, Kolkata - 700 073, India

**Keywords:** Arsenic, toxicants, arsenic and systemic manifestations, arsenic and socio-economic issues, disease burden, skin manifestations

## Abstract

**Background:**

The global health impact and disease burden due to chronic arsenic toxicity has not been well studied in West Bengal.

**Objective:**

To ascertain these, a scientific epidemiological study was carried out in a district of the state.

**Materials and Methods:**

Epidemiological study was carried out by house-to-house survey of arsenic affected villages in the district of Nadia. A stratified multi-stage design has been adopted for this survey for the selection of the participants. A total number of 2297 households of 37 arsenic affected villages in all the 17 blocks were surveyed in the district.

**Result:**

Out of 10469 participants examined, prevalence rate of arsenicosis was found to be 15.43%. Out of 0.84 million people suspected to be exposed to arsenic, 0.14 million people are estimated to be suffering from arsenicosis in the district. Highest level of arsenic in drinking water sources was found to be 1362 *μ*g/l, and in 23% cases it was above 100 *μ*g/l. Majority of the population living in the arsenic affected villages were of low socio-economic condition, inadequate education and were farmers or doing physical labour. Chronic lung disease was found in 207 (12.81%) subjects among cases and 69 (0.78%) in controls. Peripheral neuropathy was found in 257 (15.9%) cases and 136 (1.5%) controls.

**Conclusion:**

Large number of people in the district of Nadia are showing arsenical skin lesion. However, insufficient education, poverty, lack of awareness and ineffective health care support are major factors causing immense plight to severely arsenic affected people.

## Introduction

Arsenic pollution in groundwater, used for drinking purposes, has been envisaged as a problem of global concern. Arsenic contamination in drinking water has been reported from many countries like Taiwan, China, Argentina, Chile, Mexico, Cambodia, Thailand, Myanmar, Nepal, USA,(1) but the severity of this contamination in India and Bangladesh is unprecedented. The common symptoms of chronic arsenic toxicity due to prolonged drinking of arsenic contaminated water are pigmentation and keratosis and cancer of skin. In India, significant arsenic contamination in groundwater was detected in the year 1983 in West Bengal, when some villagers were diagnosed to be suffering from arsenicosis due to drinking of arsenic contaminated water. Today, in West Bengal, the arsenic contamination in ground water has been detected in 79 blocks in 8 districts of the state. Of these, the major affected districts are Malda, Murshidabad, Nadia, Burdwan and North and South 24 Parganas. It is suspected that 6 million people are exposed to arsenic contaminated ground water (>50μg/l).(1) In India, occurrences of arsenic in ground water have also been reported from Bihar, Jharkhand, Chhattisgarh, Uttar Pradesh and Assam.(1)

Over and above pigmentation, keratosis, arsenicosis produces protean manifestations like weakness, chronic respiratory disease, peripheral neuropathy, liver fibrosis, peripheral vascular disease, etc.(2,3) Remedy of chronic arsenic toxicity, known still today, is to take arsenic free water.(4,5) However, arsenic free safe water source is not available in all the arsenic affected villages of the state.

A scientific epidemiological assessment of the extent and magnitude of the problem in any region in India has not yet been made. Limited information are available regarding the disease burden due to arsenicosis in West Bengal, India. All figures quoted in various publications in regard to disease burden(6–9) are based on cases identified by scattered case detection program in the arsenic affected areas of different districts of the state. In an epidemiological survey carried out in one of the affected districts of West Bengal (South 24 Parganas), where 7683 people were examined in 57 arsenic affected villages, the prevalence of arsenical skin lesion was found to be 4.6%.(3) Further the incidence of arsenic related cancer was reported to be 5.1% among 4865 cases of arsenicosis examined during the period of 1983 to 2000.(10) However, the data of the former cross-sectional study represented information in the selected arsenic affected region of one district of the state, while the later data were compiled from cases examined in a tertiary referral centre and some scattered survey carried out by organizing various health camps and scattered examination of patients in different regions of the affected districts of the state.

**Figure 1:**
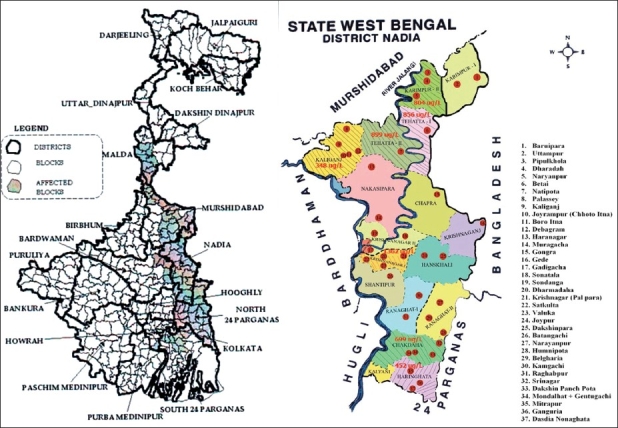
Map of West Bengal and district of Nadia with location of villages surveyed

As there has not been any authentic epidemiological study carried out in the state to estimate the disease burden of arsenicosis, a scientific epidemiological study by adopting stratified multistage design was carried out in Nadia district, one of the arsenic affected districts of West Bengal where all its 17 blocks are affected by ground water arsenic contamination [Figure 1].

## Materials and Method

### Study protocol

The epidemiological study was carried out by carrying out house-to-house survey of arsenic affected villages in the district of Nadia. Villages considered for sampling are those which have evidence of arsenic contaminated public tubewell (data obtained from survey carried out by PHED, Government of West Bengal on arsenic contaminated public tubewells in the district).(11) Census data(12) and data of arsenic contamination in public tubewells are given in Table 1. Exposed population was derived from the total population of the block as the percentage of contaminated tubewells in that block.

**Table 1:** The table presents details of Nadia District, no. of blocks and affected villages along with other relevant data on contaminated and uncontaminated tubewells, number of arsenicosis cases in the exposed population etc and estimated disease burden in the district of Nadia

Parameters	Total
Total area	8927 Sqk.m.
Total population : Ref: Census report GOI.	46,04,827
Total blocks	17
No. of villages having minimum one T.W. contaminated with As	1011
Total no. of population of blocks	3625308
Population of villages having as contamination (>50μg/l)	3220182
% of exposure (% of arsenic contaminated public tubewells in the blocks)	6.36-45.96
Total population of villages exposed to arsenic	839675
No. of villages studied	37
Total population in the study villages	635728
Total Households in the study villages	65905
No. of households surveyed	2297
Total no. of public tubewells in the district: (PHED water test report of Nadia)	29640
Total no. of public tubewell water above 50 μg/l	7662 (25.85)
No of water samples which are collected from tubewells (Govt., Pvt.) and dug wells used by villagers for drinking and cooking purpose and tested for arsenic	2116
No. of water samples contaminated with arsenic more than 50 μg/l	809 (38.23)
No. of persons examined	10469
No. of patients with arsenical skin lesion Identified	1616
Proportion of the exposed population of the blocks affected	0.04781 - 0.32249
Population derived as probable affected with arsenicosis	141592

Figures in parenthesis are percentages

### Sampling design

Outline of sample design: A stratified multi-stage design has been adopted for this survey. The first stage units (FSU) were the 2001 census villages of the arsenic affected blocks of the district. The ultimate stage units (USU) were households. Based on the scope and feasibility of the study, it was initially decided to cover 2000 households (USU) for the survey.

Sampling frame and the sample size for the First Stage Units: The villages where at least one tubewell was contaminated with arsenic at a level >50 μg/l constituted the frame for the FSUs. The data regarding arsenic contamination of tubewell water were obtained from the survey conducted by Public Health Engineering Department (PHED) of the Government of West Bengal by Spectrophotometric method. The number of such villages was 1007 out of the total of 1206 villages in the 17 affected blocks. A total of 37 villages (slightly more than 3%) constituted the sample size for this stage.

Stratification: The affected blocks were considered to be the natural geographical strata for the survey.

Allocation and selection of FSU: The number of villages allocated to each stratum was made by proportional (n_h_αN _h_) allocation depending on the number of villages in the block (stratum). The sample villages from each stratum were selected randomly with probability proportional to size (pps) method. The size referred to was the product of the total population of the village and the proportion of arsenic contaminated tubewells of the village.

Allocation of households (USU): The total number of households in the 37 selected villages was 66087. The resultant sampling fraction for the USUs came out to be 0.034 (2297/66087). Thus, the number of households selected from each sample village was about 3% of the total number of households in that village.

Selection of households (USU): Finally, the households in a sample village were selected by systematic sampling with a random start procedure after preparing a village map and listing of households.

The number of affected persons in each village was estimated in terms of the number affected among the household surveyed, assuming that the proportion affected remains the same in the households numbers not visited.

The disease burden in the district was estimated by using the formula

Disease Burden (D.B.) =

$$\sum_{i=1}^{17}N_{i}p_{i}\;P_{i}\;=\;R_{i}/n_{i}$$

where i=1 to 17

Where, N _i_ =Total population in Block i.

R _i_ =Number of patients identified in Block i.

n _i_ =Number of person examined in Block i.

### Field study

The field study was designed to minimize subjectivity in examining for skin lesions diagnostic of arsenicosis. Field workers inevitably knew if a village they were working in was located in the arsenic exposed area but they did not know the arsenic concentration in tubewells at the time of the survey. Each member of the house was interviewed and clinically examined after selection of the household in a village. Each participant was questioned briefly about his or her sources of drinking and cooking water, and duration of water use from the source. A physician took medical history from the participant. A general medical examination was carried out, including a careful inspection for arsenical skin lesions. Demographic characteristics and socio-economic condition of the participant were recorded in a proforma. All patients were exam-ined in the field by the physicians who have had about 10 years experience each in diagnosing arsenic-caused skin lesions in West Bengal, including examining patients regularly in the Arsenic Clinic in the hospital linked with the Post-graduate Medical Institute in Kolkata.

The criteria for classifying arsenical skin lesions i.e. keratosis and pigmentation were as follows: Keratosis was characterized by diffuse bilateral thickening of palms and/or soles with or without nodules of various shapes and sizes. Pigmentation was identified if there were areas of mottled dark brown pigmentation bilaterally distributed on the trunk. Sometimes spots of depigmentation also occurred, but these characteristics were not regarded as essential for the diagnosis. A scoring system has been adopted to classify the degrees of severity of skin manifestations.(5) vide Table 2, water exposure data, socio-economic characteristics and demographic features and symptomatology are compared with cases (subjects with aresnical skin lesion) and controls (subjects without arsenical skin lesion) living in the arsenic affected villages of the district.

**Table 2:** Dermatological criteria and gradation of chronic arsenic toxicity scoring system

Pigmentation (Score)		
Mild (1)	Moderate (2)	Severe (3)
Diffuse melanosis, Mild spotty pigmentation, Leucomelanosis	Moderate spotty pigmentation	Blotchy pigmentation, pigmentation of under surface of tongue, buccal mucosa
Keratosis (Score)		
Mild (1)	Moderate (2)	Severe (3)
Slight thickening, or minute papules (<2 mm) in palm and soles	Multiple raised keratosis papules (2 to 5 mm) in palm and soles with diffuse thickening	Diffuse severe thickening, large discreet or confluent keratotic elevations (>5mm), palm and soles (also dorsum of extremity and trunk)

Maximum total skin score=6; Total skin score: No skin lesion-0; Mild: 1- 2; Moderate: 3- 4; Severe:5- 6

Water samples were obtained from the tubewell on the same day as the interview and medical examinations, but results of analyses for arsenic were not known until months later. Thus, the physical examinations for skin lesions were conducted blindly without knowing water arsenic level that varied widely irregularly throughout the study region.

### Water sampling and arsenic measurement

Water samples were collected from all available current and previous private and public tubewells used for drinking and cooking purposes by each recruited household. Arsenic levels were measured by an atomic absorption spectrophotometer with flow-injection hydride generation system.

Statistical analysis: Data are reported as means ± S.D. statistical significance between groups was determined by analysis of variance with significance set at *P*<0.05.

## Results

Summary results of the study are given in Table 3. A total number of 2297 households of 37 arsenic affected villages in the 17 blocks, selected by statistically sampling method, were surveyed in the district of Nadia, West Bengal. Out of 10469 participants examined, 1,616 (15.43%) patients showed clinical features of arsenicosis (cases) characterized by arsenical skin lesion, while 8853 participants did not have any such lesion (controls). This showed a prevalence rate of arsenicosis to be 15.43%. The probable number of people affected with arsenical skin lesion in the district of Nadia appears to be 0.14 million [Table 1] as estimated by using the method of calculations described earlier.

**Table 3:** Characteristics of cases and non-cases (controls) in the district of Nadia No. of blocks: 17; No. of villages studied: 37

	Cases		Controls		*P* value
	(N=1616)		(N=8853)		
Mean peak tubewell as concentration, (μg/L)	103.469 ± 153.289		73.187 ± 115.105		<0.001
Duration of exposure to peak concentration (years)	12.477 ± 7.395		12.436 ± 6.595		<0.001
Average as exposure (ug/l)	87.56 ± 126.441		64.987 ± 101.899		<0.001
Age (Mean + S.D.):	53.36 ± 15.60		33.74. ± 15.99		
	N	%	N	%	
Sex:					
Male	934	57.80	3213	36.29	<0.001
Female	682	42.20	5640	63.71	NS
Type of dwelling:					
Kutcha	694	42.95	3842	43.40	>0.05
Kutcha-Pucca	354	21.91	2036	23.00	NS
Pucca	567	35.09	2966	33.50	NS
Sanitation status					
Absent	540	33.42	2968	33.53	>0.05
Present	1070	66.21	5852	66.10	NS
	Family (n=751)		Family (n=1546)		
Education of family head:					
Illiterate	332	44.21	520	33.64	<0.001
Primary	271	36.09	628	40.62	NS
Secondary	121	16.11	321	20.76	NS
Graduate	25	3.33	76	4.92	NS
Monthly income of family head					
<=Rs.500	42	5.59	61	3.95	<0.01
>=Rs.501-1000	321	42.74	578	37.39	NS
>Rs.1000	359	47.80	859	55.56	NS
Occupational status					
Farmer	474	63.12	860	55.63	NS
Agricultural laborer	260	34.62	680	43.98	NS
	(N=1616)		(N=8853)		
Arsenicosis skin score					
Mild (1-2 score)	1415	87.56			
Moderate (3-4 score)	187	11.57			
Severe (>4 score)	14	0.87			
Disease symptoms					
Lung disease	207	12.81	69	0.78	<0.001
Ch. Cough	127	7.86	52	0.59	<0.001
Dyspnoea	146	9.03	39	0.44	<0.001
Neuropathy (limb pain/tingling, numbness)	257	15.90	136	1.54	<0.001
Pain in abdomen	67	4.15	77	0.87	<0.001
Ch. Diarrhoea	19	1.18	15	0.17	<0.001
Liver-palpable	5	0.31	0	0.00	<0.001
Ascities	3	0.19	1	0.01	<0.05
Pallor (anemia)	1	0.06	7	0.08	>0.05
Non pitting edema of limbs	4	0.25	2	0.02	<0.01

The incidence of this disease was found to be significantly high (57.8%) in males compared to females (42.2%), (*P*<0.001) [Table 3]. The mean age was 53.36 years among cases and 33.74 years among non-cases (controls). Majority of the population living in the arsenic affected villages were of low socio-economic condition and education status, lived in kuchcha houses and were engaged in agricultural farming or physical labor. Sanitary latrine was absent in about 33% of the participants [Table 3].

Out of total number of cases having arsenical skin disease, 68.8% had only pigmentation and 1.67% had only keratosis. Both pigmentation and keratosis were found in 29.5% of the participants, majority being males. Chronic lung disease was found in 207 (12.81%) subjects among cases and 69 (0.78%) in controls ( p0 <0.001). Peripheral neuropathy and abdominal pain were found in 257 (15.9%) and 67 (4.15%) cases and 136 (1.5%) and 67 (0.87%) subjects among controls, respectively (*P* <0.001). Other systemic features found in significantly higher number of cases compared to controls are chronic diarrhoea, hepatomegaly, ascites and non-pitting edema of the limbs [Table 3]. It was interesting to observe the skin lesions to be mild (skin score-1-2) in majority (87.56%) of the cases. Though people were not aware in mild cases about their disease, severe cases with severe skin manifestations, lung disease and cancer were crippling. Because of poverty, lack of education and apathy, many of these people do not attend hospitals and many could not afford the cost of their treatment.

Mean peak arsenic level in tubewell water was 103.469 ± 153.289 μg/l in cases and 73.187 ± 115.105 μg/l in controls (*P*<0.001). Duration of exposure was about 12 years in both the groups and average arsenic exposure was 87.56 ± 126.44 μg/l in cases and 64.987 ± 101.89 μg/l in controls (*P*<0.001) [Table 3]. Water from 2116 water sources (private and public tubewells) used by all the participants were tested and value above permissible limit (50 μg/l) was found in 809 (38.23%) samples [Table 1]. Highest level of arsenic in drinking water sources consumed by the participants was found to be 1362 μg/l. However in 23% cases, the arsenic level was above 100 μg/l. In addition to contamination of private tubewells, some public tubewells and pipe water systems were also found to be contaminated with arsenic.

## Discussion

This is the first population-based study assessing the prevalence of arsenical skin lesion in a district of West Bengal, Nadia, adopting statistically valid sampling method taking into consideration level of arsenic exposure and the various socio-economic factors associated with the toxicity. Out of a population of 0.84 million people suspected to be exposed to arsenic, 0.14 million people are estimated to be suffering from arsenical skin lesion in the district [Table 1]. Earlier, attempts had been made to estimate arsenicosis disease burden in three districts of the state, Nadia, North 24 Parganas and Murshidabad, but the values derived were not based on use of statistically valid sampling method applied on the districts. Arsenic contamination in tubewells was studied in 9 blocks of Nadia district during a period of 1988-1994. According to the report, 8,922 people were found to be suffering from arsenicosis out of a population of 93624 suspected to be exposed to arsenic contaminated water in the district.(8) However, the mode of sampling of water sources for arsenic testing and method of health survey for case detection was not mentioned in the paper. In another report, on the basis of large number of water testing, 0.93 million people were suspected to be drinking arsenic contaminated water above 50 μg/l in North 24 Parganas, another arsenic affected district of West Bengal having arsenic contamination in 20 out of 22 blocks. A total of 2274 (68.9%) people were found to have arsenical skin lesion on examination of 3300 people in 62 villages of 10 blocks in that district. Further, on study of most of the population of (Kolsur) Gram Panchayat (a cluster of villages) and total population of one of its constituent village (Fakirpara) in the said district, the authors suspected that about 0.1 million people were affected with arsenical skin disease in North 24 Parganas.(13) However, the basis of this estimation is not well understood. In a separate study carried out in Murshidabad, another severely arsenic affected district of West Bengal, arsenic contamination (>50 μg/l) was found in 26% of 29,612 tubewells tested.(14) The authors estimated that probable number of arsenic exposed population in the 26 blocks of the district of Murshidabad appeared to be 1.2 million on the basis of extrapolation of data of probable water user per tubewell. Further 4813 (19%) patients had been identified to have arsenical skin lesion out of a total population of 25,274 villagers who attended health camps from 12 highly affected blocks of the said district.(15) Extrapolating all these data, probable number of people suffering from arsenicosis in Murshidabad were estimated as 0.17 million,(14) though the validity of this estimation is questionable.

In this epidemiological study in Nadia, significantly higher numbers of cases were found to be males in comparison to females. This was similar to the observation of sex distribution of arsenicosis cases in Indo-Bangla sub-continent where males were found to be more affected.(3,13,14,16–20) However, in Alashan area of Inner Mongolia, China and in Cambodia the incidences of skin lesion in males and females were found to be similar.(21,22)

Keratosis was found in 31% of the cases in this study. Keratosis has been perceived as always present in association with pigmentation.(23) However in absence of pigmentation, only keratosis was found in 1.67% of the cases in this study. This was also observed in cases of arsenicosis in Cambodia.(22) In this study, arsenical skin lesions were found to be mild in majority (87.57%) of the cases. Thus, though large number of people showed signs of arsenicosis in Nadia, most of them could be relieved of their symptom if they stop drinking arsenic contaminated water.(4) However, in many locations, safe locations of arsenic free tubewells were not available.

In the current study, chronic lung disease was found in 207 (12.81%) subjects among cases and 69 (0.78%) in controls (arsenic exposed without skin lesion). In an epidemiological study carried out in the district of South 24 Parganas 493 (11.68%) out of 4216 participants exposed to arsenic was found to have chronic lung disease.(24,25) Prevalence odd ratio in that study for non-smokers were 5.0 and 7.8 for men and women exposed to arsenic ≥ 500 μg/l compared to those exposed to <50 μg/l for participants with skin lesion, respectively, and 0.9 and 1.8 for participants without skin lesion.(24) In a village of Nawabgong district in Bangladesh, chronic cough was found in 24 (25.5%) cases out of 96 cases with skin lesion among 1237 arsenic exposed (>50 μg/l) people(16) In another study, bronchitis was found in 86 cases (23.7%) out of 363 cases of arsenicosis in a village of Jessore district in Bangladesh with a population of 3606.(17) All these data suggest that chronic arsenic toxicity causes chronic lung disease in good number of cases and these mostly occur in cases with arsenical skin lesion. In this study, peripheral neuropathy and abdominal pain were found in 257 (15.9%) and 67 (4.15%) cases and 136 (1.5%) and 67 (0.87%) subjects among controls, respectively. In earlier epidemiological studies in West Bengal(15,25,26) and Bangladesh(16,17) paresthesia and abdominal pain, chronic diarrhoea, hepatomegaly, ascites and non-pitting edema of the limbs were found in significant number of cases of chronic arsenic toxicity.

Significant difference (*P*<0.001) in peak arsenic level (103.469 ± 153.289 μg/l) was found in tubewell water in cases compared to controls (73.187 ± 115.105 μg/l). Duration of peak exposure was 12 years in both cases and controls. Average arsenic exposure was 87.56 ± 126.441 μg/l in cases and 64.987 ± 101.899 64.9 μg/l in controls and this difference was found to be significant (*P*<0.001). There was limitation of assessment of values of arsenic exposure in this study as lifetime water history could not be taken from all the cases and many of the contaminated tubewells were closed or taken out.

Most of the people studied in Nadia were farmers or agricultural laborers and belonged to low socio-economic class. A significant number of people lived in kuchcha house had low earning and literacy and did not have sanitary latrine. Further there was not much significant difference in these socio-economic characteristics among cases and controls. Though similar observations were reported in regard to occupation and socio-economic class among arsenic exposed population in West Bengal and Bangladesh,(9,27–30) few data were available regarding these characteristics among cases and controls. Not much significant difference in socio-economic characteristics has been observed among cases and controls in this study and in similar study done in South 24 Parganas.(31)

Since arsenicosis was found to occur mostly among poor and usually less educated people, it has had far reaching social consequences. Many public water sources including piped water supply system were found to be contaminated with arsenic in this study. Though some seriously arsenic affected people including cancer patients gave history of attending arsenic referral centers at Kolkata, they failed to get admission because of paucity of beds. Further, inability to pay cost of investigation, treatment and bed rent in those hospitals prevented admission in hospitals and caused immense suffering and death of many severely ill poor arsenicosis patients. As there is no early perceptible symptom or sign of arsenic toxicity and the people are less educated, they are reluctant to test their water sources for arsenic and continue to drink arsenic contaminated water. Social counseling need to be given to the people in the community, to avoid social stigmatization of women and children, with arsenic-induced skin lesions. They need to be convinced that arsenical skin disease is neither infectious nor hereditary and hence need not be segregated from others. Due to a general lack of awareness, people suffer silently fearing ridicule and ostracism. Victims rarely report disease early. The impact on the livelihood of people remains underestimated. The various clinical manifestations of arsenicosis are crippling. The progress of the disease is accompanied by deteriorating health and directly affects the earning ability of adults, thus further lowering economic status of the family.

## Conclusion and Recommendation

A scientific epidemiological assessment of the extent and magnitude of the problem of chronic arsenic toxicity has not yet been made in any region in India earlier. On the basis of cross-sectional study done on 2297 households of 37 arsenic affected villages in all the 17 blocks in the district of Nadia, West Bengal, selected by statistically sampling method, prevalence rate of arsenicosis was found to be 15.43% out of 10469 participants examined. The probable number of people affected with arsenical skin lesion in the district appears to be 0.14 million. Majority of the population living in the arsenic affected villages were of low socio-economic condition, inadequate education and were engaged in agricultural farming or physical labour. It was interesting to observe the skin lesions to be mild in majority (87.5%) of the cases. Hence, supply of arsenic free water will help in amelioration of symptoms in these people. However, about 38% of the water sources, some of which are public tubewells and piped water supply system, were found to be contaminated with arsenic in the district. It is therefore an urgent need to make arrangement for availability of safe water source among the arsenic affected people in the district. Many of the people in the affected villages are not aware of contamination of their home tubewells with arsenic. Awareness generation and motivation of the people for testing their drinking water sources for arsenic are also important to prevent further exposure of arsenic to these people.

Further, arsenic affected people with severe skin lesions and systemic manifestations like lung disease, neuropathy etc are having unbearable suffering. These people are very poor and live in distant villages where hospital facilities are not easily available. Arrangement for free treatment of these patients in state referral hospitals and free transport facility from their villages could help a lot in alleviating the suffering of these people.
